# Investigating ocular biomarkers and differential diagnosis of Alzheimer’s disease and vascular cognitive impairment based on multimodal imaging

**DOI:** 10.1117/1.JBO.29.12.126003

**Published:** 2024-12-27

**Authors:** Zi Jin, Xuhui Chen, Chunxia Jiang, Ximeng Feng, Kun Shang, Jinying Li, Qiushi Ren, Chuanqing Zhou

**Affiliations:** a Eye Hospital, Wenzhou Medical University, National Engineering Research Center of Ophthalmology and Optometry, Wenzhou, China; b Eye Hospital, Wenzhou Medical University, National Clinical Research Center for Ocular Diseases, Wenzhou, China; c Peking University Shenzhen Graduate School, Institute of Biomedical Engineering, Shenzhen, China; d Institute of Biomedical Engineering, Shenzhen Bay Laboratory, Shenzhen, China; e Peking University Shenzhen Hospital, Department of Neurology, Shenzhen, China; f Peking University Shenzhen Hospital, Department of Ophthalmology, Shenzhen, China; g Peking University, College of Future Technology, Department of Biomedical Engineering, Beijing, China; h Shanghai University of Medicine and Health Sciences, College of Medical Instruments, Shanghai, China

**Keywords:** Alzheimer’s disease, vascular cognitive impairment, multimodal ophthalmic imaging, pupillary light reaction, retinal reflectance spectrum, retinal hemodynamics

## Abstract

**Significance:**

The eye can be used as a potential monitoring window for screening, diagnosis, and monitoring of neurological diseases. Alzheimer’s disease (AD) and vascular cognitive impairment (VCI) are common causes of cognitive impairment and may share many similarities in ocular signs. Multimodal ophthalmic imaging is a technology to quantify pupillary light reaction, retinal reflectance spectrum, and hemodynamics. This provides multidimensional ocular metrics from a non-invasive approach to ocular biomarkers and differential diagnosis of AD and VCI.

**Aim:**

We aim to investigate the changing pattern of ocular metrics in patients with AD and VCI using multimodal ophthalmic imaging.

**Approach:**

Patients with subjective cognitive complaints in the memory clinic were subdivided into AD, VCI, and cognitively healthy individuals using neuropsychological and neuroimaging examinations, including positron emission tomography. All subjects underwent a medical history review, blood pressure measurement, medical optometry, intraocular pressure measurement, and custom-built multimodal ophthalmic imaging. Multidimensional parameters were analyzed by one-way analysis of variance and *post hoc* comparisons.

**Results:**

This study included 19 patients with AD, 24 patients with VCI, and 37 cognitively healthy age- and sex-matched subjects. Both AD and VCI patients showed abnormal pupillary light reactions, including decreased resting pupil diameter, pupil constriction amplitude, and maximum constriction velocity. Compared with the control group, the AD group presented increased retinal reflectance at 548 nm, whereas the VCI group presented an increased resistivity index and decreased blowout score in retinal hemodynamics.

**Conclusions:**

We demonstrate that pupillary light reaction-related neurodegeneration is the common pathological change in both AD and VCI. In addition, AD is characterized by alterations in retinal spectral signatures, whereas VCI is characterized by alterations in retinal hemodynamics. These findings suggest that multimodal ophthalmic imaging may have the potential to be used as a screening tool for detecting AD and VCI.

## 1 Introduction

Given the rapid global aging trend, the prevalence of cognitive impairment is escalating rapidly, posing an urgent public health concern.^1^ The primary causes of cognitive impairment are Alzheimer’s disease (AD) and vascular cognitive impairment (VCI), respectively.^2^^,^^3^ According to the diagnostic criteria, AD is a cognitive disorder due to the deposition of core pathological proteins such as amyloid beta (Aβ) and phosphorylated tau proteins, whereas VCI is a cognitive disorder due to cerebrovascular disease.^4^^,^^5^ The clinical symptoms of AD and VCI are similar, including cognition disorders and mental symptoms, which are easily confused in clinics.^2^^,^^3^ Neuropsychological examination reveals that AD cognitive deficits are more frequently characterized by impairments in amnestic and parietal functions, whereas VCI is more often associated with attentional and executive dysfunction.^6^ Pathologically, AD is pathologically characterized by the presence of Aβ plaques, phosphorylated tau, neurofibrillary tangles, and cerebrovascular amyloidosis, whereas VCI is pathologically characterized as ischemic, hypoperfusion, and hemorrhagic damage.^4^^,^^7^ Therefore, it is crucial to distinguish between AD and VCI to guide treatment and evaluate the prognosis of cognitive impairment for more appropriate treatment. Currently, neuroimaging tests are used to identify AD and VCI, but the method is time-consuming and expensive.^8^ The eye is considered as the window of the brain, and ocular examination is an objective, quick, and low-cost alternative. As a result, ocular examination has recently become a research focus for detecting cognitive impairment.^1^^,^^9^^–^^13^

Nowadays, several types of ocular changes have been reported in patients with cognitive impairment.^9^^,^^10^ First, several groups have found AD patients and other-cause cognitive impairment have an abnormal pupillary light reaction (PLR) with reduced velocity and amplitude of constriction, which may be caused by the nervous dysfunctions in the retina and/or the brain.^14^^–^^16^ Second, both AD and VCI have been reported to exhibit abnormal retinal hemodynamics, including reduced retinal blood speed and blood flow.^17^^–^^19^ Third, the retinal spectral signature acquired through retinal spectral imaging has been demonstrated to label retinal biomarkers of AD and shows promising performance in predicting preclinical AD patients.^20^^,^^21^ The retinal spectral signature appears to have the potential to distinguish between AD and VCI. To date, most published studies have focused on a singular type of cognitive impairment, with minimal attention given to identify AD and VCI through ophthalmic imaging.

Because AD and VCI may have many similarities in ocular signs, a single modal ophthalmic imaging will not be sufficient to distinguish them.^9^^,^^22^ Thus, it is necessary to apply multimodal ophthalmic imaging to accurately detect and differentiate AD and VCI.^11^^,^^22^ Recently, we have developed a custom-built multimodal ophthalmic imaging device to assess PLR, retinal hemodynamics, and retinal spectral reflectance simultaneously.^23^^,^^24^ In the present work, we first investigated the alterations of PLR, retinal hemodynamics, and retinal spectral reflectance among AD, VCI, and cognitively healthy age- and sex-matched subjects. To the best of our knowledge, this study will provide new insights into the identification of AD and VCI using ophthalmic imaging.

## 2 Materials and Methods

### 2.1 Subjects and Study Procedure

This study was approved by the Institutional Review Board of Human Research at the Ethics Committee of Peking University Shenzhen Hospital (Approval ID: 2021-004 (Research) -01). Informed consent was obtained from each subject, and all procedures were followed in accordance with the Declaration of Helsinki.

From February 2021 to February 2022, 19 AD patients and 24 VCI patients were diagnosed in this study by a neurologist (X.C.) in the Memory Disorders Clinic. All patients underwent a clinical history review, Montreal Cognitive Assessment (MoCA), and neuroimaging tests. MoCA is a widely used overall neuropsychological evaluation tool for detecting cognitive impairment, of which the MoCA total score is lower than 27 points.^25^ According to the National Institute on Aging-Alzheimer’s Association (NIA-AA) criteria, AD was diagnosed on the basis of brain biomarkers of AD using cerebrospinal fluid biochemistry or positron emission tomography.^4^ VCI was diagnosed based on the presence of white matter hyperintensities, ischemia, or hemorrhage on magnetic resonance imaging.^26^ In addition, 37 cognitively healthy age- and sex-matched subjects aged ≥50 years were recruited from the neurology or ophthalmology clinic. Control subjects had no subjective memory complaints and normal cognitive test results. Exclusion criteria included a history of glaucoma, age-related macular degeneration, severe visual impairment (corrected visual acuity less than 0.3), and any medical conditions that might preclude fundus examination.

Two experienced performers took the neurology and ophthalmology evaluation for all subjects, including medical history, blood pressure measurement, MOCA, best-corrected visual acuity (BCVA), intraocular pressure (IOP), and multimodal ophthalmic imaging. First, an automated oscillometric device (Kenz AC-05C, SUZUKEN CO., LTD., Aichi, Japan) was used to obtain systolic and diastolic blood pressure (SBP, DBP). Second, autorefraction (ARk-1, NIDEK CO., LTD., Aichi, Japan) and subjective refraction were performed successively for each subject. Third, the eye with better BCVA was selected for multimodal ophthalmic imaging. Finally, a non-contact tonometer (TX-20, Canon Inc., Tokyo, Japan) was used to measure the IOP of both eyes.

The custom-built multimodal ophthalmic imaging device has been described in detail previously.^23^ In brief, the device was modified from a regular nonmydriatic fundus camera and integrated with different imaging techniques such as PLR, retinal multispectral imaging (MSI), and laser speckle contrast imaging (LSCI). Retinal MSI can obtain the retinal spectral reflectance, and LSCI can measure retinal hemodynamics. With this device, we performed PLR first, followed by LSCI and MSI for each individual in the dim room without mydriasis. More detailed information on multimodal ophthalmic imaging was described as follows.

#### 2.1.1 Measurement of PLR

Subjects were asked to fixate on the target within the device and try their best to keep their eyes open for a 12-s measured period. The flash stimulus was presented at the 3rd second and lasted 500 ms in the measured period. If participants blink during the light stimulation, the measurement will be repeated. The pupil segmentation algorithm based on the threshold was used to obtain pupil diameter from captured pupil images.^27^ Therefore, the pupil diameter dynamic variation to light was obtained and PLR-related parameters were calculated for further analysis, including resting pupil diameter (RPD), latency time (LAT), constriction amplitude (CA), relative constriction amplitude (RCA), average constriction velocity (ACV), and maximum constriction velocity (MCV).

#### 2.1.2 Measurement of retinal hemodynamics

Each subject was asked to fixate on the target within the device and avoid blinking during a 5-s measured period for capturing retinal speckle pattern images. The retinal blood velocity was calculated with a custom-developed LSCI algorithm.^23^ The optic nerve head area in the retinal perfusion image was chosen to obtain the retinal blood flow pulsatility curve. According to the pulse-waveform analysis, we obtained the retinal hemodynamics parameters, including flow acceleration index (FAI), blowout score (BOS), and resistivity index (RI).^28^

#### 2.1.3 Measurement of retinal reflectance spectra

Each subject was asked to fixate on the target within the device and avoid blinking in MSI using five wavelengths (808, 740, 660, 600, and 548 nm) in sequence. Exposure time was set to 0.03 s per frame, making it possible to obtain good registration among frames.

We chose an area in the superior of temporal vascular arcades above the macula rather than overall images to analyze the retinal reflectance spectra because the reflectance spectra have the greatest difference at locations superior to the temporal vascular arcades above the macula between AD and control.^20^ The spectral reflectance can be calculated by the following formula.^20^

$$R_{\lambda }=\mathrm{Log}\;10(\frac{I_{\lambda }}{C_{\lambda }\times I_{0}|\lambda }),$$ (1)

where $R_{\lambda }$ is the spectral reflectance of the wavelength λ, $I_{\lambda }$ is the intensity in retinal multispectral images of the wavelength λ, $I_{0}|\lambda$ is the light power of the wavelength λ measured in the corneal plane, and $C_{\lambda }$ is the response coefficient of the camera for the wavelength λ.

Because the pupil diameter has an influence on the retinal spectral reflectance,^29^ we adjusted Eq. (1) when the pupil diameter is less than the threshold. The threshold of pupil diameter is set to 4 mm, and the correction equation is as follows.

$$RC_{\lambda }=\frac{R_{\lambda }}{\min(p,4)}\times 4,$$ (2)

where $RC_{\lambda }$ is the spectral reflectance of the wavelength λ after correction and p is the pupil diameter.

### 2.2 Data Analysis

Data analysis was performed using SPSS software version 19.0 (IBM Corp., New York, United States) and Matlab 2019b (MathWorks, Natick, United States). Continuous variables were analyzed by one-way analysis of variance and Bonferroni post hoc tests, and categorical variables were analyzed by the chi-square test. A value of P<0.05 was considered statistically significant.

## 3 Results

The basic demographics of the three groups are summarized in Table 1. There was no significant difference in basic demographics among the three groups, except for MOCA. The MOCA scores in AD and VCI were significantly lower than in controls, whereas there was no significant difference in the MOCA score between AD and VCI.

**Table 1:** Clinical characteristics of subjects among AD, VCI, and control.

	Control (n=7)	VCI (n=24)	AD (n=19)	P
Age, years	65.4 (5.8)	67.7 (8.4)	66.6 (8.6)	0.49
Gender (M/F)	15/22	8/16	8/11	0.80
Diabetes, no. (%)	5 (13.5)	5 (20.8)	2 (10.5)	0.61
Hypertension, no. (%)	10 (27.0)	8 (33.3)	5 (26.3)	0.84
SBP, mmHg	124.1 (20.2)	120.6 (17.3)	130.3 (15.5)	0.32
DBP, mmHg	75.0 (13.7)	72.9 (7.9)	79.0 (10.4)	0.32
BCVA	4.97 (0.08)	4.92 (0.10)	4.94 (0.09)	0.18
SE, D	0.01 (1.11)	0.11 (0.88)	0.00 (1.35)	0.94
IOP, mmHg	13.6 (2.0)	13.6 (2.6)	12.5 (2.3)	0.26
MOCA score	**28.0 (1.5)**	**15.4 (7.0)**	**12.8 (6.1)**	**<0.01**

**Abbreviations:** AD, Alzheimer’s disease; BCVA, best-corrected vision acuity; DBP, diastolic blood pressure; SE, spherical equivalent; IOP, intraocular pressure; MOCA, Montreal Cognitive Assessment; SBP, systolic blood pressure; VCI, vascular cognitive impairment. Bolded text represents results that are statistically different between groups.

**Fig. 1:**
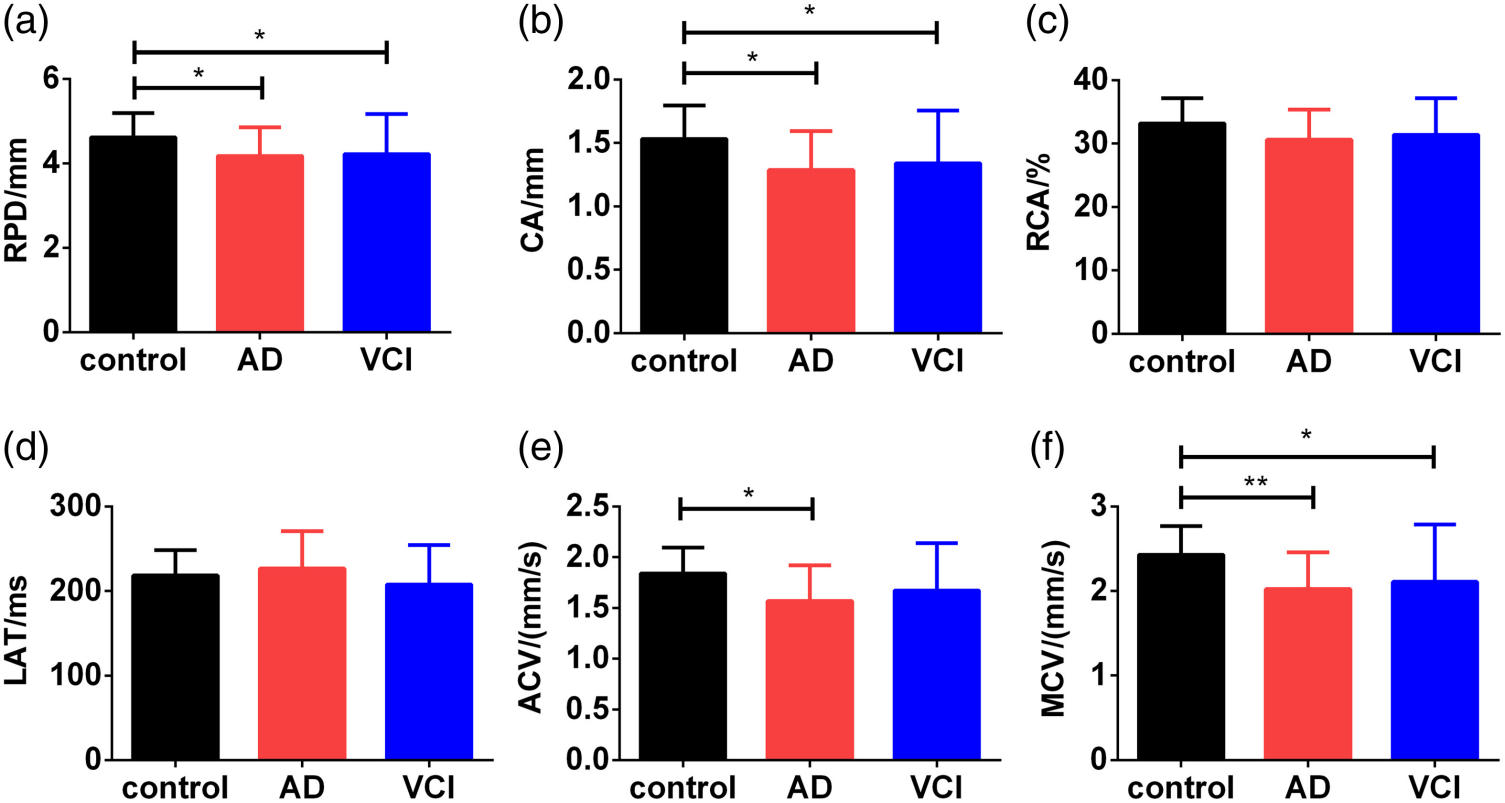
Comparison of the parameters of PLR among controls, AD, and VCI. ACV, average constriction velocity; AD, Alzheimer’s disease; CA, constriction amplitude; LAT, latency time; MCV, maximum constriction velocity; RCA, relative constriction amplitude; RPD, resting pupil diameter; VCI, vascular cognitive impairment. *p<0.05; **p<0.01.

Compared with the control group, significant differences in PLR were found in both AD and VCI groups. Both AD and VCI groups exhibited reduced RPD, CA, and MCV, whereas the LAT and RCA did not change significantly [Figs. 1(a)–1(d) and 1(f)]. In addition, the AD group showed decreased ACV, whereas the VCI group only had a decreased trend compared to the control group [Fig. 1(e)].

**Fig. 2:**
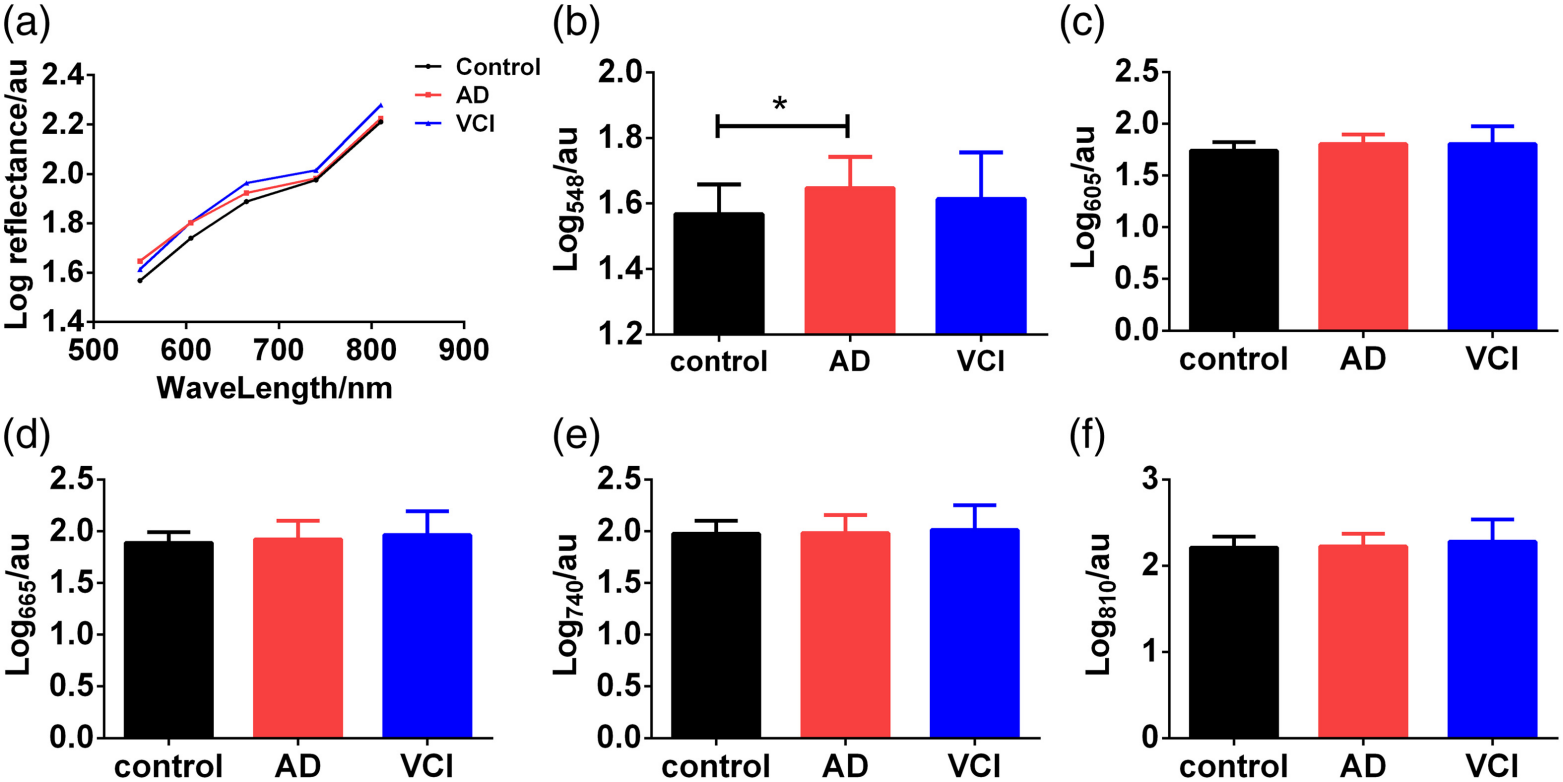
Comparison of the parameters of retinal reflectance spectra among controls, AD, and VCI. (a) The retinal reflectance spectra curve between 548 and 810 nm. (b)–(f) Results of retinal reflectance spectra among three groups at 548, 605, 665, 740, and 810 nm, respectively. AD, Alzheimer’s disease; VCI, vascular cognitive impairment. *p<0.05.

Figure 2(a) shows the retinal reflectance spectra curves of controls, AD, and VCI. Interestingly, only retinal reflectance spectra at 548 nm increased significantly in the AD group compared with the control group, whereas the reflectance spectra of other wavelengths did not change significantly [Figs. 2(b)–2(f)].

**Fig. 3:**
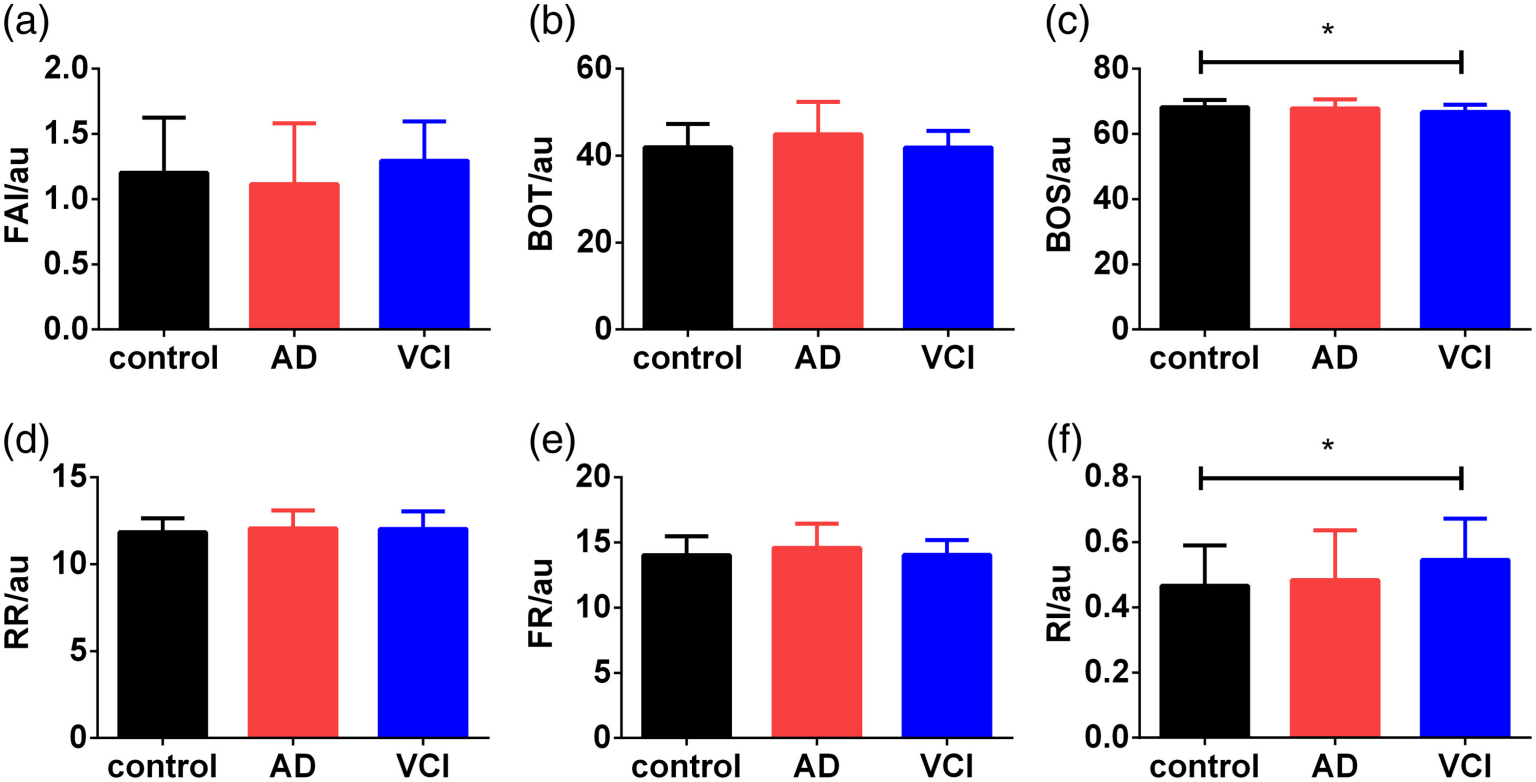
Comparison of the parameters of retinal hemodynamics among controls, AD, and VCI. AD, Alzheimer’s disease; BOT, blowout time; BOS, blowout score; FAI, flow acceleration index; FR, falling rate; RI, resistance index; RR, raising rate; VCI, vascular cognitive impairment. *p<0.05.

Figure 3 provides the summary statistics for retinal hemodynamics among controls, AD, and VCI. It is worth noting that there were significant differences in the BOS and RI for the VCI group but not for the AD group compared with the control group.

Table 2 presents the specific results of the ocular biomarkers measured by multimodal imaging among three groups.

**Table 2:** Summarized multidimensional ocular metrics results of subjects among AD, VCI, and control.

	Control (n=37)	VCI (n=24)	AD (n=19)
**PLR-related parameters**			
RPD, mm	**4.62 (0.58)** ^a^ ^,^ ^b^	**4.22 (0.95)** ^c^	**4.18 (0.68)** ^c^
CA, mm	**1.53 (0.26)** ^a^ ^,^ ^b^	**1.34 (0.42)** ^c^	**1.28 (0.31)** ^c^
RCA, %	33.2 (4.0)	31.4 (5.8)	30.6 (4.7)
LAT, ms	218.4 (30.0)	207.9 (46.8)	226.5 (44.3)
ACV, mm/s	**1.84 (0.26)** ^b^	1.67 (0.47)	**1.57 (0.35)** ^c^
MCV, mm/s	**2.43 (0.34)** ^a^ ^,^ ^b^	**2.11 (0.68)** ^c^	**2.02 (0.44)** ^c^
**Retinal reflectance-related parameters**			
Log retinal reflectance spectra at 548 nm	**1.57 (0.09)** ^b^	1.61 (0.14)	**1.64 (0.10)** ^c^
Log retinal reflectance spectra at 605 nm	1.74 (0.08)	1.80 (0.17)	1.80 (0.09)
Log retinal reflectance spectra at 665 nm	1.89 (0.10)	1.96 (0.23)	1.92 (0.18)
Log retinal reflectance spectra at 740 nm	1.98 (0.13)	2.01 (0.24)	1.99 (0.17)
Log retinal reflectance spectra at 810 nm	2.21 (0.13)	2.28 (0.26)	2.22 (0.14)
**Retinal hemodynamics-related parameters**			
FAI	1.20 (0.42)	1.29 (0.30)	1.11 (0.47)
BOT	41.9 (5.3)	41.9 (3.8)	44.9 (7.5)
BOS	**68.2 (2.2)** ^a^	**66.7 (2.3)** ^c^	67.8 (2.84)
RR	11.8 (0.8)	12.0 (1.0)	12.0 (1.0)
FR	14.0 (1.4)	14.0 (1.2)	14.6 (1.8)
RI	**0.47 (0.12)** ^a^	**0.54 (0.13)** ^c^	0.48 (0.15)

ACV, average constriction velocity; AD, Alzheimer’s disease; BOT, blowout time; BOS, blowout score; CA, constriction amplitude; FAI, flow acceleration index; FR, falling rate; LAT, latency time; MCV, maximum constriction velocity; RCA, relative constriction amplitude; RI, resistance index; RPD, resting pupil diameter; RR, raising rate; VCI, vascular cognitive impairment. Bolded text represents results that are statistically different between groups.

a Statistical difference with the VCI group.

b Statistical difference with the AD group.

c Statistical difference with the control group.

## 4 Discussion

The purpose of this study is to identify AD and VCI using ophthalmic imaging. To our knowledge, the present work is the first to apply the retinal spectral signatures, neurological function, and hemodynamics together in patients with AD and VCI. Compared to a matched control group, both AD and VCI patients show significant differences in the PLR, which suggests PLR-related neurodegeneration is a common pathological change. Furthermore, AD patients show abnormal retinal spectral signatures, whereas VCI patients show abnormal retinal hemodynamics. Thus, the multimodal ophthalmic parameters will facilitate the detection of cognitive impairment and may even enable the identification of AD and VCI.

In this study, PLR has demonstrated good discrimination in cognitive impairment, independent of AD and VCI patients. Previously, most publications reported abnormal PLR in AD patients, including reduced constriction amplitude and velocity,^30^^–^^33^ which is similar to our results in AD. Parkinson’s disease patients with cognitive impairment also present similar change patterns in PLR with AD.^16^ In contrast, there has been little research investigating the effect of VCI on PLR. In terms of the structure of neural reflex arcs, PLR is affected by pathological changes in both the retina and the brain.^14^ Obviously, both AD and VCI show brain pathological changes, including functional network changes and brain atrophy.^34^ In addition, retinal nerve fibers and ganglion cells involved in PLR undergo degeneration in the AD and VCI.^9^ In summary, these common nervous pathological changes may contribute to the abnormal change in PLR.

Another important finding is that the retinal reflectance at 548 nm increases significantly in the AD group, whereas it does not change significantly in the VCI group. Using retinal hyperspectral imaging, several groups have reported remarkably distinguishable reflectance in the spectral range of 450 to 550 nm between AD and control subjects, similar to our results.^20^^,^^21^^,^^35^^,^^36^ In the retina, small soluble Aβ oligomers and phosphorylated tau may contribute to the short visible wavelength spectral signatures in AD.^1^^,^^21^ As these deposits are biomarkers of AD, it is understandable that the AD group has significantly increased retinal reflectance at 548 nm. The results suggest that retinal MSI contributes to the differential detection of AD and VCI.

The results of this study show that retinal hemodynamic changes were observed only in the VCI group, but not in the AD group. Specifically, RI increased and BOS decreased significantly in the VCI group. RI and BOS are valid parameters to characterize retinal angiopathy. RI is calculated by dividing the difference between the maximum and minimum blood flow velocity by the maximum blood flow velocity, and BOS indicates the volume of blood flow maintained in the vessel during each heartbeat.^28^ Using transcranial Doppler ultrasonography, patients with VCI are found to have more severe disturbances in cerebral hemodynamics compared to AD.^37^ Several reports have shown that patients with VCI have a higher pulsatility index (PI) and lower cerebral blood flow velocity compared with age-matched healthy controls. It has been reported that cerebral PI and RI are the most valuable for the diagnosis of VCI compared with maximum velocity, minimum velocity, and mean velocity of cerebral blood flow.^38^ As far as we know, this is the first time to report retinal RI and BOS change significantly in VCI. Several previous studies have reported a decrease in blood flow in patients with mild cognitive impairment (MCI) and probable AD.^17^^,^^18^^,^^39^ According to AD biomarkers, Olafsdottir et al. divided MCI into AD and MCI lacking clear biomarkers for AD and found the latter have more severe retinal angiopathy disturbances compared to AD, which is similar to our findings.^40^ A possible explanation for this might be that VCI has a more pronounced disturbance in retinal hemodynamics than AD, similar to the alteration in cerebral hemodynamics of VCI and AD. Because MCI without clear biomarkers for AD may include VCI, it is easily understood that individuals with MCI lacking clear biomarkers for AD exhibit significant changes in retinal oxygen metabolism due to retinal angiopathy.^41^ Based on the definitions of BOS, blood flow, RI, and PI, BOS has a positive relationship with blood flow and a negative relationship with RI and PI.^28^ Thus, increased RI and decreased BOS in our study suggest decreased blood flow and increased PI, consistent with previous results in cerebral and retinal hemodynamics. The results of our study suggest that retinal hemodynamics may also aid in the differential detection of AD and VCI.

There are several limitations in this study. First, the sample size of our study is relatively small, which may have an influence on the results. Second, this study excludes patients with known ocular diseases. It is known that many ocular diseases, such as glaucoma and age-related macular degeneration, can affect the PLR, retinal reflectance spectra, and retinal hemodynamic results.^1^ Thus, further work is required to confirm the impact of ocular diseases on the detection of AD and VCI. Third, the VCI and control groups did not have a biomarker test for AD, so it is possible that both groups in this study may include AD individuals with normal or impaired cognition, which may affect the results. It has been reported the frequency of Aβ positivity in VCI and cognitively healthy individuals ranges from 30% to 53%, and 18% to 26%, respectively.^34^^,^^42^ Based on the above data, most of the subjects in VCI and control groups are not mixed with AD, so the results obtained are relatively reliable. Finally, the healthy subjects are recruited from hospitals and may suffer from other diseases other than AD and VCI. Hospitalized individuals might have different health characteristics compared with the general public; thus, generalizing the results of this study to the general population should be with caution.

## 5 Conclusion

The present study demonstrates that PLR-related neurodegeneration is a common pathological change in AD and VCI, whereas alterations in retinal spectral signatures and retinal hemodynamics appear to be specific to AD and VCI, respectively. These findings provide a theoretical basis for the detection of AD and VCI using multimodal ophthalmic imaging. Through this study, the fundamental understanding of the changes pattern of ocular multidimensional metrics in both AD and VCI can be advanced, which will further contribute to the development of eye-brain association research.

## Data Availability

Data underlying the results presented in this paper are not publicly available at this time but may be obtained from the authors upon reasonable request.
